# Zwitterionic Coatings for Urinary Catheters: Current Progress, Design Principles and Translational Challenges

**DOI:** 10.1021/acsomega.6c07621

**Published:** 2026-10-09

**Authors:** Chenghao Yao, Zhengda Wang, Sixu Yan, Shuai Zhang

**Affiliations:** School of Pharmacy, 1596 Queen’s University Belfast, Lisburn Road, Belfast BT9 7BL, U.K.

## Abstract

Catheter-associated urinary tract infection (CAUTI) is one of the most common healthcare-associated infections and remains a major cause of morbidity, catheter blockage, and repeated device replacement during long-term catheterization. This narrative review examines the interfacial processes that contribute to urinary catheter failure, including protein adsorption, microbial adhesion, biofilm formation, encrustation, and swarming-associated blockage, and evaluates how zwitterionic surface coatings may address these challenges. Drawing primarily on urinary catheter-specific studies, together with adjacent zwitterionic biomaterials literature where relevant to catheter design, this review synthesizes current coating strategies with emphasis on protein repellence, lubrication, biocompatibility, multifunctionalization, interface-specific design requirements, architectural trade-offs between hydrogel-like and brush-like coatings, and approaches to improve coating durability. The review also considers translation, including interim human evidence for zwitterion-treated urinary catheters, while recognizing that these data remain conference-reported and product-specific. Overall, zwitterionic coatings should be viewed not as a single antifouling solution, but as a versatile biomaterials platform for engineering multifunctional, durable, and clinically translatable urinary catheters. However, their long-term stability, resistance to complex biofilm and encrustation processes, and clinical efficacy require further validation. To define these remaining validation needs, the review compares PC-, SB-, and CB-based systems, stratifies evidence by model and duration, reports quantitative coating data where available, and identifies validation requirements across the product lifecycle.

## Graphical abstract

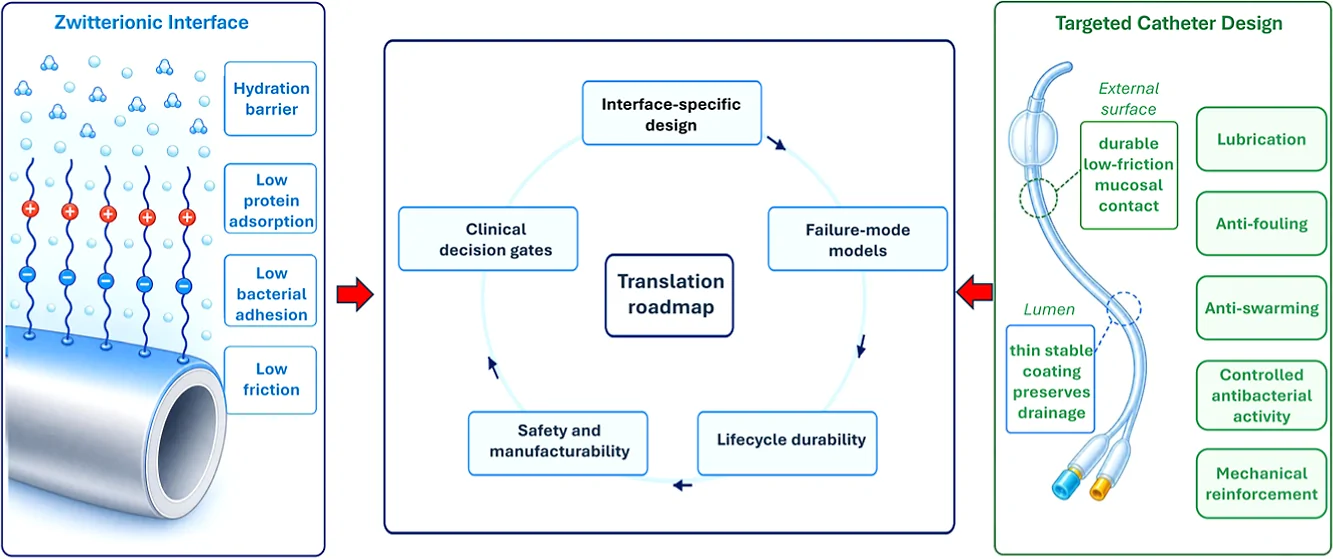

## 1 Introduction

### 1.1 Urinary Catheters as an Interfacial Problem

Urinary catheters remain among the most frequently used medical devices in hospitals, with approximately 15–25% of patients undergoing catheterization and more than 30 million catheters used annually in the United States alone.^1,2^ Driven by population aging and the growing reliance on acute and chronic bladder management, the global urinary catheter market is expanding rapidly and has been forecast to grow at an annual growth rate of 6.69% from 2026 to 2033.^3^ Clinically, urinary catheterization enables reliable bladder drainage when spontaneous voiding is impaired and supports perioperative management, neurogenic bladder dysfunction, urinary retention, and selected palliative settings.^1,4,5^ Yet once inserted, a catheter is not simply a passive conduit for urine. It becomes a biomaterial interface exposed simultaneously to urine, host proteins, microbes, and the mechanically sensitive urethral mucosa.^5,6^ For that reason, catheter performance depends not only on drainage but also on how effectively the device limits tissue injury, host-protein conditioning, microbial attachment, and intraluminal fouling. Catheter-associated urinary tract infection (CAUTI), traumatic catheterization, and blockage should therefore be viewed not as isolated complications (Figure 1), but as coupled outcomes arising at the catheter-urine, catheter-mucosa, and catheter–lumen interfaces.^7–9^

**Figure 1:**
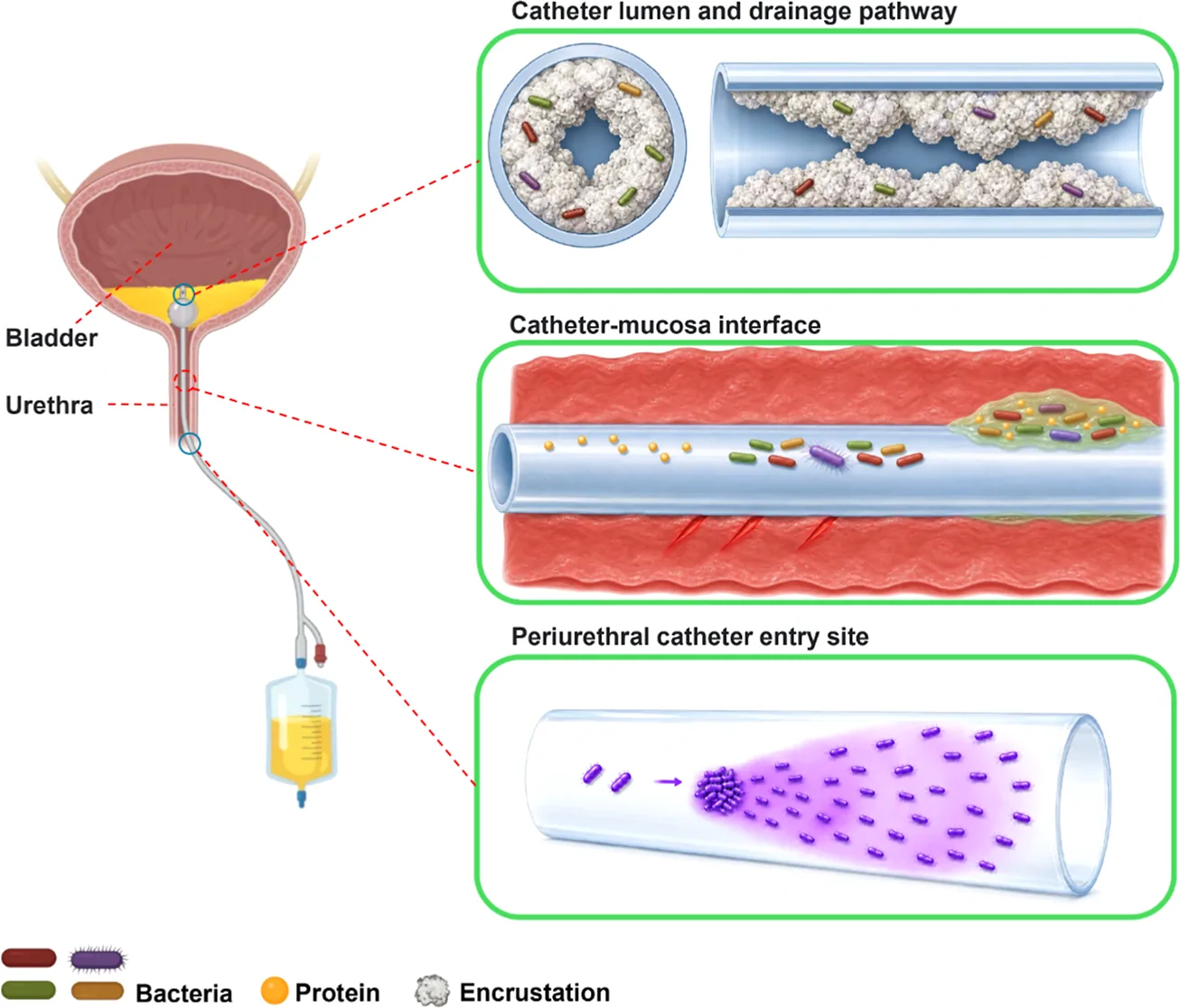
Schematic representation of key interfacial failure sites associated with indwelling urinary catheterization, including friction-induced urethral microtrauma, mucosal irritation at the catheter–mucosa interface, protein adsorption, periurethral bacterial colonization and ascending migration, biofilm formation, and crystalline encrustation within the catheter lumen and drainage pathway. Created by the authors using Microsoft PowerPoint (Microsoft 365).

This interfacial view becomes more important with prolonged dwell time. Long-term catheterization is associated with progressive bacteriuria or colonization, recurrent symptomatic infection, blockage, bypassing, and repeated device exchange, with the risk of colonization increasing by approximately 3–7% per catheter day and approaching near-universal colonization during chronic use.^10–13^ At the same time, most commercial catheters are manufactured from hydrophobic materials such as silicone, latex, polyvinyl chloride, and polyurethane, which are not inherently well matched to the urethral mucosa.^14^ Repeated insertion, indwelling contact, and withdrawal can therefore produce frictional injury and microtrauma. Recent *ex vivo* porcine work further links catheter withdrawal force to urothelial membrane damage, reinforcing the idea that tribological performance affects tissue injury directly.^15^ Once injury occurs, the host response promotes deposition of proteins such as fibrin and fibrinogen on the catheter surface.^16^ These deposits form a conditioning layer that facilitates bacterial adhesion and biofilm development, making trauma and infection mechanistically linked rather than independent failure modes.

Once colonization is established, long-term CAUTI also differs microbiologically from uncomplicated UTI. In chronically catheterized patients, biofilms are frequently polymicrobial and may persist across repeated catheter exchanges.^17–19^ Cooperative interactions within these communities increase persistence and treatment tolerance, while the catheter surface itself shelters organisms from both host clearance and antimicrobial exposure.^19,20^ A further escalation of failure occurs when urease-producing organisms, especially *Proteus mirabilis* (*P. mirabilis*), raise urinary pH, drive precipitation of calcium and magnesium phosphates, and generate crystalline biofilms that progressively obstruct the catheter lumen.^9,21–23^ Therefore, long-term catheter failure is best understood as an interfacial cascade comprising frictional injury, host-protein conditioning, microbial adhesion, biofilm maturation, and, in many patients, biomineralization and obstruction.

### 1.2 Limitations of Current Catheter Coatings

To date, existing strategies for catheter surface modification can be broadly grouped into antimicrobial surfaces, which aim to reduce viable organisms at the device interface, and antifouling or lubricious surfaces, which aim to prevent the earliest steps of trauma, conditioning, and microbial attachment.^6,24^ Both categories have generated important advances. However, neither has yet produced a universally satisfactory long-term solution for indwelling urinary catheters because each tends to solve only part of the failure cascade (Table 1).

**Table 1:** Existing Urinary Catheter Coating Landscape as a Comparative Framework

coating class	examples	main strength	long-term limitation	zwitterionic relevance
silver-based antiseptic coatings	silver-alloy/metal catheters^25,27^	some short-term bacteriuria reduction	limited/inconsistent symptomatic CAUTI benefit, especially long-term^25,27^	killing alone is not durable protection
nitrofurazone/nitrofural release coatings	drug-impregnated catheters^26,27^	can delay early bacteriuria	finite payload, urine-dependent activity, discomfort, and weak durability^26,27^	shows limits of release-only designs
lubricious coatings	PTFE, PVP hydrogel, PEG-related hydrogel-coated catheters^33,35–38^	better handling, comfort, and mucosal tolerance	lubricity alone rarely lowers indwelling CAUTI; drying, adhesion, and swelling remain concerns^33,35–38^	comfort helps but does not control infection
other antimicrobial-release coatings	antibiotics, metal ions, phages, peptides, enzymes^55–60^	broad options against cells or biofilm	burst/depletion, diffusion, urine stability, resistance/toxicity, and durability concerns^28,30–34^	favors nondepleting base plus optional payloads
stimuli-responsive antimicrobial coatings	pH-, light-, or trigger-activated interfaces^33^	temporal control; lower unnecessary exposure	trigger reliability, light access, pH variation, durability, and *in vivo* gaps^30–33^	zwitterions supply continuous baseline antifouling
passive low-fouling hydrophilic/brush coatings	PEG-like coatings, polymer brushes, electroneutral surfaces^24,61–63^	targets conditioning films; antibiotic-sparing	depends on grafting density, conformation, substrate, and urine-specific durability^24,61–63^	benchmarks hydration-driven low fouling
dynamic antiadhesion interfaces	liquid-infused, liquid-like, and superhydrophobic coatings^16,39–42^	strong preclinical antibiofilm/antiencrustation effects	durability, lubricant retention, and long-term urinary translation remain concerns^16,39–42,46–49^	dynamic interfaces must also be stable
antibiofilm/swarming polymers	high-throughput discovery-derived materials and antiswarming catheter coatings^43–45^	targets *P. mirabilis* swarming and crystalline biofilm	sparse clinical validation; device translation still developing^43–45^	supports urine-tailored multifunctional zwitterions

For antimicrobial coatings, the best-studied clinical examples remain silver-alloy and nitrofurazone or nitrofural catheters. These systems can reduce bacteriuria in some short-term settings, yet benefits for symptomatic infection are limited or inconsistent, and evidence from short-term Foley use cannot be extrapolated straightforwardly to long-term catheterization.^25–27^ More broadly, release-based or contact-active antimicrobial coatings face recurring translational constraints, including finite payload, altered activity in urine, masking by host-protein deposition, variable performance under flow, sterilization and toxicity concerns, manufacturing complexity, and tension with antimicrobial stewardship.^7,13,16,27–34^ Antimicrobial killing on its own therefore often intervenes only after microorganisms have already reached the device, while its protective effect may diminish over clinically relevant dwell times.

Antifouling coatings aim to prevent early protein and bacterial adhesion, but maintaining their performance over extended catheterization is challenging. In practice, these coatings often lose efficacy under chronic, polymicrobial biofilm conditions, which limits their long-term benefit. Commercial polytetrafluoroethylene (PTFE) and hydrogel-based coatings, particularly polyvinylpyrrolidone (PVP)- and polyethylene glycol (PEG)-based systems, remain clinically important because they improve lubricity and mucosal tolerance, especially in intermittent catheterization.^33,35–38^ However, their anti-infective benefit in indwelling Foley use has generally remained modest and context dependent. Newer dynamic platforms, including liquid-infused surfaces, superhydrophobic interfaces, and high-throughput discovery-derived polymers resistant to biofilm formation or swarming, have shown strong preclinical performance against fibrinogen accumulation, bacterial retention, or crystalline attachment.^16,39–45^ Even so, most remain validated mainly in simplified *in vitro* or short-duration *ex vivo* models, and many nonbiocidal systems may lose effectiveness once stable colonization is established under persistent polymicrobial challenge.^46–49^

Thus, the current coating literature suggests that the main difficulty is not simply the absence of surface activity, but the challenge of integrating several requirements within one catheter interface, including resistance to host-protein conditioning, durable lubrication, dimensional fidelity within the lumen, stability under urine exposure and wet–dry cycling, control of swarming and encrustation, realistic manufacturability, and persistence across the intended dwell period.^13,16,33,46–49^ This unresolved combination of interfacial, mechanical, microbiological, and translational requirements creates the rationale for focusing on zwitterionic systems.

### 1.3 Zwitterionic Coatings as a Potential Solution to Current Challenges

Zwitterionic materials are worth discussing not as another generic entry in the coating catalogue, but as a distinct interfacial design platform. Their charge-balanced structures generate strong hydration, which can create a highly hydrated barrier to nonspecific adsorption while also supporting lubrication and biocompatibility.^50–54^ Relative to conventional hydrophilic coatings, this raises the possibility of encoding function directly at the interface rather than relying solely on a thick lubricious film or a finite antimicrobial payload. Just as importantly for urinary devices, zwitterionic chemistries can be deployed as thin grafted layers, brushes, films, or hydrogel-like networks, allowing the coating architecture to be tailored to the distinct requirements of catheter outer surfaces and luminal interfaces.

This narrative review focuses specifically on zwitterionic coatings for urinary catheters, with the aims of summarizing their chemical and interfacial basis, evaluating current evidence for their ability to prevent CAUTI and related complications, and identifying the principal barriers to translation, particularly with respect to durability, clinically relevant performance, and manufacturability. Literature selection prioritized peer-reviewed original research articles and review papers published between 2018 and 2026 that were directly relevant to zwitterionic urinary catheter coatings. Adjacent biomaterials studies were also included when they provided direct mechanistic or translational insight relevant to catheter design. Given the heterogeneity in coating chemistries, test models, and end points, the evidence is synthesized qualitatively rather than quantitatively, and no formal meta-analysis or structured risk-of-bias assessment was performed.

To complement prior reviews that survey antimicrobial and antifouling catheter coatings broadly, the present review is organized around the catheter failure cascade and interface-specific design constraints rather than as a chemistry catalogue.^6,24,33,50^ This organization addresses three questions: (1) which failure step and catheter surface is targeted; (2) how chemistry and architecture balance hydration, dimensional fidelity, and mechanical stability; and (3) whether the available model and duration support a claim limited to early antiadhesion, prolonged preclinical protection, or patient-level clinical benefit. Table 2 then compares representative systems quantitatively, while treating evidence gaps as design constraints rather than assuming equivalence.

**Table 2:** Representative Zwitterionic Urinary Catheter Coatings: Design, Quantitative Performance, Evidence Duration, and Limitations^a^

chemistry and representative system	substrate/coating method/thickness	mechanism and key quantitative outcomes	test model/duration/durability	major limitation and evidence level
PC: poly(MPC-*co*-HEMA-*co*-BP) (PMHB)^64^	polyurethane urinary catheter; pregel spin coating followed by UV curing; thickness NR	hydration-mediated lubrication and antifouling. After 7 d, reported antibacterial rates were 72.98% (*E. coli*), 75.11% (*S. aureus*), and 88.23% (*P. mirabilis*); cell viability was >95%	7 d bacterial coincubation and 7 d artificial-urine cycling; reduced encrustation attachment. Quantitative coating adhesion and poststerilization performance NR	promising 7 d preclinical PC evidence. No polymicrobial flow or clinical study; exact coefficient of friction and thickness NR
SB: PSBMA hydrogel^65^	PVC; surface photopolymerization; approximately 8 μm coating thickness	hydration-mediated antifouling/lubrication. Fibrinogen deposition decreased 72.3%; *E. coli* and *S. aureus* adhesion decreased >75%; dynamic coefficient of friction decreased from 0.63 to 0.35	1 h fibrinogen exposure; 24 h static and up to 72 h flow bacterial adhesion; short-duration *P. mirabilis* crystalline-biofilm assessment	strong early comparative evidence, but passive and nonbiocidal. Chronic polymicrobial, *in vivo*, cyclic-adhesion, sterilization, and aging data NR
SB copolymer: PSBMA-*co*-PHPA (PSH)^66^	PDMS catheter material; silane-assisted free-radical coating; thickness NR	SB hydration plus HPA-mediated antiswarming and reduced swelling. PSBMA equilibrium swelling was approximately 510%; HPA incorporation reduced swelling by up to 91%. Static/dynamic coefficients of friction were 0.15/0.10 (PSBMA) and 0.29/0.27 (PHPA). The 4:1 SBMA/HPA coating reduced fibrinogen by approximately 40% and biofilm coverage approximately 4-fold versus PDMS	bacterial and swarming tests up to 7 d; no visible delamination after 4 w in water	clear formulation trade-off: more HPA improves swelling control/antiswarming but weakens antifouling. Preclinical; no polymicrobial, sterilization, or *in vivo* validation
SB: polydopamine/EGDMA-anchored PSBMA^67^	PDMS and commercial silicone urinary catheter; polydopamine interlayer, EGDMA linker, and *in situ* SBMA polymerization; thickness NR	hydration-mediated bacteriophobicity. *E. coli* adhesion decreased by 1–2 orders of magnitude (90–99%) on PDMS and by >99% on the commercial catheter under the reported condition; PDMS water contact angle decreased from 115 ± 3° to 19 ± 6°	approximately 18 h adhesion and 50 h repeated-feed biofilm tests; repeated loading, stretching, torsion, and clamping showed no delamination	useful device-shape and mechanical-challenge evidence, but no standardized coating–substrate fracture energy, cyclic insertion, urinary mineralization, sterilization, or clinical data
SB/QAC multifunctional polyzwitterion with cellobiose dehydrogenase^68^	PDMS catheter material; electrostatic layer-by-layer assembly (six layers); thickness NR	zwitterionic antifouling plus QAC contact killing and enzyme-generated H_2_O_2_. Copolymers contained up to 50% zwitterionic groups; bacterial attachment decreased by up to 60% with evidence of killing	short-duration *in vitro* bacterial tests; multilayer stability assessed on model surfaces by ellipsometry, contact angle, zeta potential, and AFM. Long-term urinary duration NR	mechanistically informative multifunctional proof-of-concept. Layer-by-layer complexity, limited duration, and no urine-flow, encrustation, sterilization, or *in vivo* data
CB/QAC: QPEI with poly(carboxybetaine-*co*-dopamine methacrylamide) (QCB)^69^	urinary catheter; mussel-inspired codeposition of bactericidal QPEI and adhesive CB copolymer; thickness NR	CB antifouling plus cationic contact killing. Reported bacterial antiadhesion was >99.5% and killing efficiency >99.9%	daily bacterial exposure for 30 d *in vitro* with prevention of biofilm formation; rabbit urinary-tract infection model supported *in vivo* activity	strong prolonged preclinical evidence, but the antifouling–cationic balance, quantitative adhesion, sterilization/aging, and patient outcomes remain unresolved
CB/Ag composite: poly(carboxybetaine) with silver-phenolated lignin nanoparticles^70^	silicone urinary catheter; laccase-assisted *in situ* nanoparticle grafting and CB polymerization; thickness NR	CB-mediated protein resistance plus nonantibiotic silver bactericidal activity. Surface bacterial viability decreased by >2 log under hydrodynamic flow; no apparent cytotoxicity was reported	function maintained for 1 w *in vitro* and *in vivo*; animal models indicated biocompatibility	promising week-scale preclinical evidence. Silver release/depletion, long-term encrustation, mechanical adhesion, sterilization/aging, and clinical durability remain unresolved
proprietary zwitterion-treated catheter platform (ClearTract)^71–75^	medical-grade silicone catheter; commercial surface-treatment chemistry, method, architecture, and thickness not publicly reported in the cited sources	antibiofouling surface treatment. Interim conference data (NCT04841226): mean ATP mass on explanted catheter surfaces, used as a surrogate for active biomass, was 137.07 ng/in (*n* = 36), versus 337.29 ng/in (*n* = 38) for silicone-coated and 316.80 ng/in (*n* = 40) for silver-coated catheters	randomized multicenter postmarket study (114 patients; up to 30 d). Ongoing crossover at the reported data cut (NCT05931887): 7 infections/131 visits and 53.4 mg encrustation versus 22/130 visits and 169.2 mg during standard-of-care periods; 24/25 questionnaire respondents preferred the treated catheter	the only human outcome evidence identified in this review remains interim, conference-reported, and product-specific, with no full peer-reviewed trial report available. Evidence from one proprietary platform with undisclosed chemistry and processing cannot establish class-wide efficacy for PC, SB, or CB coatings

a Note: NR indicates that a value was not reported in the cited study or source. Values are reproduced as reported, but direct comparison is inappropriate because substrate, thickness state, conditioning medium, organism, loading, urine formulation, flow, and end point differ. Reports of qualitative coating integrity or the absence of visible delamination are distinguished from quantitative measurements of coating–substrate adhesion.

## 2 Zwitterionic Coatings for Urinary Catheters

### 2.1 Zwitterionic Materials

#### 2.1.1 Chemistry and Interfacial Properties

Zwitterionic polymers are defined by the presence of both cationic and anionic groups within the same repeat unit, yielding overall charge neutrality at the macroscopic scale while maintaining a highly polar local structure.^50^ Following the early development of polysulfobetaines and the later introduction of phosphorylcholine-based polymers such as poly­(2-methacryloyloxyethyl phosphorylcholine) (PMPC), these materials became the basis of a broader family of charge-balanced biomaterials.^51^ The principal antifouling zwitterionic polymer classes include phosphorylcholine (PC), sulfobetaine (SB), and carboxybetaine (CB) polymers, which typically combine quaternary ammonium cations with phosphate, sulfonate, or carboxylate anions, respectively.^50^ This molecular arrangement distinguishes polyzwitterions from polyampholytes or mixed polyelectrolytes, in which opposite charges are distributed across different repeat units and therefore do not achieve the same degree of molecular-scale charge compensation.^52^ A key consequence of this chemistry is electrostatically induced hydration, or ionic solvation, whereby zwitterionic materials bind water strongly and generate a dense hydration shell that acts as both a physical and energetic barrier to nonspecific adsorption.^53^ The same hydration mechanism also contributes to other interfacial functions relevant to biomaterials design, including lubrication and, in some systems, enhanced ion transport or biomolecular stabilization.^54,76–78^ For urinary catheters, this hydration–rich interface is attractive because it may simultaneously reduce conditioning-film formation, support low-friction surface contact, and remain compatible with thin surface-bound architectures.

This molecular architecture confers several advantages over more established antifouling materials, particularly PEG-based systems and conventional hydrogels.^79^ Although PEG has historically served as a benchmark nonfouling polymer, its long-term biomedical use is constrained by oxidative degradation,^24^ thermal instability,^80^ dependence on chain conformation and coating density,^81^ and increasing concern regarding immunogenicity.^82^ By contrast, zwitterionic materials resist nonspecific adsorption through ionic solvation rather than hydrogen-bond-mediated hydration and often maintain a strong and persistent hydration layer under physiologically relevant conditions and across a broad range of salt concentrations and temperatures.^50^ Current evidence further suggests that hydration, rather than simple wettability alone, is a major determinant of nonfouling performance.^83^ In practice, the tightly associated interfacial water layer functions as an energetic barrier because proteins, cells, and microorganisms must displace bound water before attachment can occur. Consistent with this view, even rigid zwitterionic monolayers can strongly resist protein adsorption when dense interfacial hydration is maintained, and spectroscopic and thermal analyses indicate that zwitterionic materials retain strongly associated water while perturbing bulk water structure less than many conventional polymers.^53,84^

These interfacial properties have supported the deployment of zwitterionic materials across a range of biomedical contexts where biofouling, thrombosis, or foreign body reaction limit performance, including blood-contacting devices,^85,86^ implantable sensors,^87^ cell-encapsulation systems,^88^ ophthalmic materials,^89,90^ and selected bioelectronic interfaces.^91,92^ The relevance of these broader examples is not that urinary catheters are identical to them, but that they demonstrate how zwitterionic interfaces can combine low fouling, lubrication, and biocompatibility in surface-bound formats. For urinary catheters specifically, the central implication is that zwitterionic chemistries offer a flexible interfacial platform that can, in principle, integrate protein repellence, lubricity, and biocompatibility within a single surface design.

#### 2.1.2 Chemistry-Dependent Implications for Urinary Catheters

These shared interfacial advantages do not make PC, SB, and CB interchangeable. Phosphorylcholine (PC) polymers such as PMPC present a phosphate/quaternary-ammonium pair that mimics the outer headgroup of phospholipid membranes. Both charges remain constitutive over the clinically relevant urinary pH range, supporting stable hydration and low nonspecific adsorption without requiring ionization of a weak acid.^50,53^ For catheter coatings, PC is attractive where biocompatibility and lubrication must be combined in thin or cross-linked interfaces. However, catheter-specific evidence is less extensive than for SB in the studies reviewed here. The PMPC-containing photocurable coating in ref 64 supports urinary feasibility, but head-to-head durability data against SB and CB coatings on the same catheter substrate are unavailable.^64^

#### 2.1.3 Sulfobetaine

SB polymers combine a sulfonate with quaternary ammonium and, like PC, remain zwitterionic over urinary pH. Among the three families, SB provides the broadest catheter-specific preclinical evidence in the literature reviewed here, including reductions in protein/bacterial adhesion and friction, antiswarming copolymers, and thin anchored or multilayer systems.^65–68^ That same strong hydration, however, can become an architectural liability: ionic screening in saline or urine can disrupt inter- and intrachain ion pairing and produce antipolyelectrolyte expansion.^50,53^ In thick or weakly cross-linked networks, this can increase swelling, softening, and interfacial stress. Cross-linking, hydrophobic comonomers, and covalent anchoring can improve stability, but may also reduce hydration or antifouling. Composition and thickness must therefore be optimized together.

#### 2.1.4 Carboxybetaine

In contrast to PC and SB, CB polymers replace sulfonate or phosphate with a carboxylate, enabling postfunctionalization and, depending on structure and local p*K*_a_, pH-responsive modulation of charge. When the carboxylate remains deprotonated, charge balance and hydration support low fouling. In sufficiently acidic microenvironments, however, protonation reduces net neutrality and can be exploited for charge switching, while also making performance more environment-sensitive.^50,53^ Current urinary evidence is concentrated in multifunctional CB coatings rather than standardized head-to-head comparisons.^69,70^ These differences do not justify an intrinsic PC > SB > CB ranking. PC offers biomimetic, pH-independent hydration; SB currently has the strongest catheter-specific preclinical coverage but can show salt-responsive swelling; and CB offers useful functionalization/responsive chemistry with greater potential environmental sensitivity. Accordingly, each chemistry must be evaluated with its architecture and anchoring in artificial and human urine spanning relevant pH, ionic strength, flow, and dwell time.

### 2.2 Mechanistic Rationale for Zwitterionic Coatings in Preventing CAUTI and Related Complications

The mechanistic appeal of zwitterionic coatings becomes clearest when CAUTI is viewed as a surface-conditioned, trauma-amplified process rather than a purely microbiological event. In short-term catheterization, early events such as urethral and bladder microtrauma, catheter-surface conditioning, and initial bacterial adhesion help determine whether bacteriuria and infection develop. In long-term catheterization, the same cascade is compounded by polymicrobial persistence, *P. mirabilis* swarming, urease-driven alkalinization, and crystalline encrustation, ultimately leading to blockage, recurrent infection, and device failure.^16,44,93,94^ Within this framework, the central question is not simply whether a coating is nonfouling, but whether it addresses the specific interfacial requirement operating at each stage of failure: microtrauma calls for durable lubrication; early conditioning calls for resistance to protein deposition; luminal use demands low-profile interfaces that preserve geometry; and long-term indwelling use increasingly requires strategies against swarming, persistent biofilm formation, and encrustation. Zwitterionic coatings are attractive in this context because their hydration-mediated interface may counteract several of these early interfacial events simultaneously.

Because the catheter failure cascade develops over time, this review treats exposure duration and model complexity as separate dimensions of evidence. Protein adsorption and bacterial-attachment tests lasting ≤24–48 h are classified as initial-interface evidence; multiday single-species studies as intermediate preclinical evidence; and ≥7 day dynamic models that incorporate urine chemistry, flow, polymicrobial challenge, urease/mineralization, or device deformation as more relevant preclinical tests of prolonged protection, while remaining distinct from clinical evidence.^13,16,44,65,93,94^ A longer exposure alone does not establish relevance to long-term catheterization if these features are absent. Accordingly, reduced early attachment supports a claim of early antifouling, but not protection against chronic polymicrobial biofilm, encrustation, blockage, or symptomatic CAUTI. These evidence categories are analytical labels used in this review, not clinical definitions of short- versus long-term catheterization.

This principle is illustrated by a direct comparison of four advanced antifouling urinary catheter surfaces, in which a PSBMA coating outperformed liquid-like, liquid-infused, and superhydrophobic alternatives in fibrinogen adsorption, biofilm development, and early crystalline deposition.^65^ The same hydrated interface may also improve boundary lubrication, making zwitterionic coatings plausible candidates for intervening early in the CAUTI cascade. This potential advantage becomes more relevant when the limitations of conventional hydrogel coatings are considered. The performance of classical lubricious coatings such as PVP is often transient because these surfaces can dry out rapidly and become increasingly mucoadhesive or “sticky,” thereby increasing the risk of epithelial damage and coating delamination during withdrawal.^95^ In a biomimetic urethral model, most PVP-coated catheters caused substantial urothelial cell loss after only 2 min of contact, consistent with surface dry-out during clinically relevant dwell time.^96^ This limitation matters in both short- and long-term use because drying between insertion and withdrawal can convert a lubricious coating into a source of adhesion and microtrauma, while repeated cycling is likely to compromise performance consistency further.^97^ By contrast, zwitterionic coatings rely on a more stable hydration state than thick, highly swollen lubricious films and may therefore be better positioned to retain function during repeated wet–dry and insertion–withdrawal cycles, although broader device-level validation remains necessary.

This mechanistic logic also has an architectural consequence. The strong wetting and interfacial activity of zwitterionic materials make them especially amenable to micro- and nanoscale coating formats, including self-assembled layers, thin grafted films, and polymer brushes.^50,98,99^ Unlike many conventional hydrogels, which often require relatively thick and highly swollen coatings to achieve lubrication and are therefore more vulnerable to swelling and delamination, zwitterionic brushes and other ultrathin grafted structures can in principle deliver a hydration barrier with minimal added thickness.^100^ This feature is particularly relevant to catheter lumens, where preservation of surface conformity and lumen geometry is essential. In this sense, zwitterionic design offers not only a chemical advantage but also a route toward lower–profile interfaces better matched to urinary catheter constraints.

Zwitterionic coatings also differ fundamentally from traditional antimicrobial coatings. Silver-, nitrofural-, and antibiotic-based catheters are designed to suppress infection after microorganisms have already reached the device, but in practice their effects are often modest or transient,^101^ and their release-based mechanisms are constrained by finite local payload and declining effective exposure over time.^102^ Zwitterionic surfaces instead function primarily as antiadhesive interfaces that intervene earlier in pathogenesis without relying on sustained antimicrobial elution, making them conceptually aligned with antibiotic-sparing interfacial strategies and avoiding the same direct drug selection pressure imposed by eluting antimicrobial systems. However, once colonization becomes established, passive antifouling alone may no longer be sufficient. In one recent modeling study, short-term catheterization outcomes were predicted to depend more strongly on catheter-surface properties, whereas long-term outcomes became increasingly sensitive to urinary and bladder parameters such as urine production rate and residual bladder volume.^13^ Thus, these considerations support zwitterionic coatings as a strong platform for suppressing the earliest stages of CAUTI pathogenesis while also providing a versatile foundation for multifunctional design against the more complex biological cascade that emerges during long-term catheterization.

### 2.3 Clinical and Translational Evidence for Zwitterionic Urinary Catheters

In contrast to the extensive mechanistic and preclinical literature, current human evidence for zwitterionic urinary catheters remains limited and is centered on a single commercial platform, the 2-way 100% Silicone ClearTract catheter.^71^ Official regulatory documents indicate that the device lineage includes the original HDX Silicone 2-Way Foley Catheter clearance under K192034, a subsequent ClearTract relabeling/IFU update under K221625, and a later K222118 update extending the indications for suprapubic and nephrostomy placement.^72–74^ These documents support an accurate description of the device as a single-use, medical-grade silicone Foley catheter with a surface modification and labeled indwelling use for up to 30 days, but they should not be interpreted as demonstrating clinical superiority in patient-important outcomes.

Against this regulatory background, the only human outcome evidence identified in the cited literature comes from a 2024 conference abstract/ePoster,^75^ which reports interim results from two randomized studies rather than providing a full peer-reviewed trial publication. The first, a prospective randomized multicenter postmarket study (NCT04841226), enrolled 114 patients who received a study catheter for up to 30 days. Mean ATP mass on explanted catheter surfaces, used as a surrogate for active biomass, was 137.07 ng/in for the zwitterion-treated group (*n* = 36), compared with 337.29 ng/in for the silicone-coated group (*n* = 38) and 316.80 ng/in for the silver-coated group (*n* = 40). Because ATP mass is a device-level surrogate, this result does not directly establish a patient-important outcome. The second was a randomized crossover study (NCT05931887) that remained in progress at the reported data cut. During zwitterion-treated periods, 7 catheter infections were reported across 131 patient visits and a mean encrustation mass of 53.4 mg, compared with 22 infections across 130 visits and 169.2 mg during standard-of-care periods; 24 of 25 questionnaire respondents preferred the zwitterion-treated catheter. Taken together, these findings are encouraging, but they remain interim, conference-reported, and product-specific. Consequently, they cannot establish class-wide clinical efficacy or rank PC, SB, and CB chemistries.

### 2.4 Recent Research Advances in Zwitterionic Coatings for Urinary Catheters

#### 2.4.1 From Nonfouling Proof-of-Concept to Multifunctional Urinary Interfaces

Recent advances in zwitterionic coatings for urinary catheters reflect a clear shift from generic nonfouling proof-of-concept toward the design of multifunctional urinary interfaces. Early studies mainly treated zwitterionic materials as passive antiadhesive coatings that reduce protein adsorption and bacterial attachment through hydration-mediated surface resistance. That objective remains important, but it is now too narrow to capture the broader causes of catheter failure, which increasingly appear as a coupled interfacial cascade involving microtrauma, host-protein conditioning, bacterial colonization, swarming, polymicrobial biofilm formation, and encrustation.^103^ Current research therefore asks not only whether zwitterionic hydration can suppress early fouling in principle, but also how that hydrated interface can be adapted to the distinct mechanical and microbiological demands of urinary catheter use. A useful organizing concept in this literature is the trade-off between hydration-mediated function and structural robustness: the same strong hydration that supports lubrication and antifouling can also complicate long-term mechanical stability, especially as coating thickness increases. Where examples are drawn from adjacent device classes or noncatheter polymer systems, they are best interpreted as design analogs rather than direct urinary catheter validation.

#### 2.4.2 Lubrication and Trauma Control

Lubrication is a rational first design priority because catheter insertion is one of the earliest clinically relevant device-host events, and excessive friction at this stage can injure the urothelium, amplify local inflammation, and promote the host-protein deposition that later facilitates bacterial colonization.^104,105^ In this sense, lubricity is not merely a comfort-related attribute, but an upstream determinant of fouling and infection risk. For urinary catheters, however, the design implications of lubrication are surface-specific rather than uniform across the device. The outer surface ideally requires durable lubrication to minimize microtrauma during insertion, indwelling, and withdrawal, whereas the inner luminal surface must preserve urine flow and catheter geometry, making thick lubricious layers less attractive. This surface-specific logic is also consistent with CDC/HICPAC guidance recommending the smallest bore catheter consistent with good drainage to minimize urethral trauma and noting that silicone may be preferable in long-term catheterized patients with frequent obstruction from encrustation.^105^ Outer-surface lubrication and inner-lumen dimensional fidelity may therefore need to be optimized separately rather than through a uniform whole-device coating.

Accordingly, zwitterionic coatings may offer advantages over conventional hydrophilic coatings because they provide lubrication through a tightly bound hydration layer encoded directly at the interface, rather than through a thick, highly swollen film that may lose performance after dehydration or repeated wet–dry cycling. However, compared with antifouling design, long-lasting lubrication in zwitterionic urinary catheter coatings has received less direct attention. Most current efforts instead focus on stabilizing the hydrated interface without sacrificing mechanical integrity, given the intrinsic trade-off between strong hydration and weak mechanical stability. The main strategies have been to enhance coating-substrate attachment through covalent grafting^64^ or to incorporate hydrophobic components into the zwitterionic network to restrain swelling.^43^ In each case, the goal is to preserve hydration-mediated lubrication under urine flow and repeated mechanical contact while limiting coating delamination, excessive swelling, encrustation, and biofilm formation. Related concepts have also been explored in adjacent biomedical settings through gradient and double-network architectures,^106,107^ and high-density polymer brushes,^108,109^ all directed toward maintaining a stable lubricious interface under mechanically and chemically challenging conditions.

#### 2.4.3 Synergistic Antiadhesion, Antiswarming, and Antibacterial Design

A second major direction in the field has been the extension of classical antifouling design toward urinary specific biological challenges, particularly *P. mirabilis* swarming and subsequent encrustation. In urinary catheters, resistance to early protein deposition and bacterial attachment is necessary but often insufficient because *P. mirabilis* can subsequently differentiate into hyperflagellated swarmer cells, migrate along the catheter surface, and drive ascending infection together with crystalline biofilm formation.^44^ This limitation is illustrated by work showing that a pure zwitterionic hydrogel coating, although highly effective in reducing fibrinogen adsorption and early biofilm formation, remained vulnerable to swarming and showed weak long-term water stability.^65^ The field has therefore moved beyond passive low-fouling design alone toward coatings that either suppress migration without directly killing bacteria or combine antiadhesive function with bactericidal or responsive behavior.

One instructive nonbiocidal example is the poly­(sulfobetaine methacrylate-*co*-2-hydroxy-3-phenoxypropyl acrylate) (PSBMA-*co*-PHPA) coating (Figure 2A), in which the zwitterionic PSBMA phase provides hydration-mediated lubrication and resistance to fibrinogen deposition, whereas PHPA functions as a swarming-inhibitory component within a urinary catheter coating designed to suppress swarming-associated failure.^66^ The broader significance of this study lies less in the specific copolymer formulation than in the design principle it illustrates: hydration-mediated antifouling can be retained while an additional material component is introduced to address a urinary-specific failure mode. Importantly, the same formulation also improved water stability relative to more conventional zwitterionic hydrogels, again underscoring the recurring tension between functional hydration and structural robustness.

**Figure 2:**
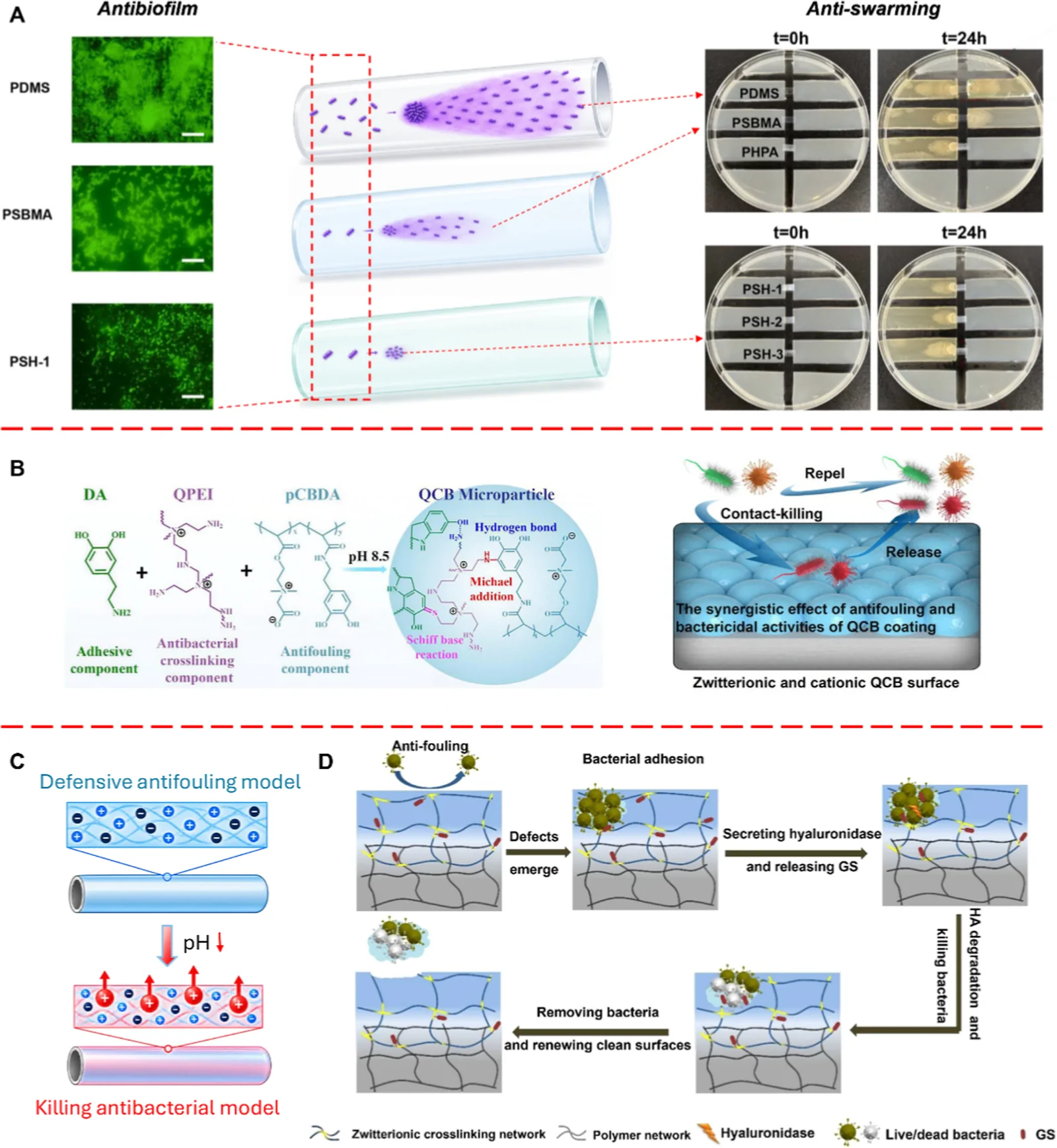
Antiadhesion and antibacterial zwitterionic coating strategies for urinary catheter applications. (A) Zwitterionic copolymer coatings with synergistic antibiofilm and antiswarming properties. Reproduced with permission from ref 66. Copyright 2025 Elsevier. (B) Quaternary ammonium moieties-immobilized zwitterionic coatings with synergistic antifouling and contact-killing activities. Reproduced with permission from ref 69. Copyright 2023 Elsevier. (C) pH-responsive zwitterionic coatings maintain an antifouling state under physiological conditions but switch to an antibacterial mode in infection-associated microenvironments, as reported in ref 113. Schematic created by the authors. (D) Self-defensive zwitterionic hydrogel coatings enable bacteria-triggered drug release, surface degradation, and interfacial renewal, converting bacterial fouling into a recoverable antibacterial and antifouling response. Reproduced from ref 114. Copyright 2022 American Chemical Society. The figure layout and annotations were assembled by the authors using Microsoft PowerPoint (Microsoft 365).

A parallel strategy has been to integrate bactericidal function into the zwitterionic matrix. One route combines hydration-mediated antifouling with immobilized contact-killing groups, most commonly quaternary ammonium motifs. Representative examples include urinary catheter coatings in which quaternary ammonium polyethylenimine is integrated with zwitterionic carboxybetaine-containing layers^69^ (Figure 2B), and related sulfobetaine/quaternary ammonium copolymers.^110^ These systems follow a straightforward but important design logic: the zwitterionic component reduces nonspecific adhesion, while the cationic component kills adherent bacteria. Their principal advantage is that they avoid burst release and may provide more sustained local activity. Their limitations are equally clear: increasing cationic content may improve bactericidal potency, but often at the cost of greater protein adsorption and cytotoxicity.^111,112^ Hydration and killing therefore do not increase together indefinitely, but must be balanced compositionally.

This trade-off is inherently nonmonotonic. Contact killing requires cationic groups to remain accessible at the urine–surface interface, whereas low fouling requires dense charge balance and a persistent hydration layer. Raising the quaternary-ammonium fraction can improve membrane disruption but can also increase protein/cell attraction, hemolysis, or cytotoxicity; conversely, excessive zwitterionic shielding can prevent bacteria from contacting the cationic domains.^69,110–112^ Accordingly, the design target is the minimum accessible cationic density that retains killing after urine/protein conditioning without measurably sacrificing lubrication, antifouling, or biocompatibility. Evaluation should therefore report surface cationic density and leaching, include zwitterion-only and cationic-only controls, and repeat killing and antifouling assays after sequential microbial challenges. On this basis, current urinary systems provide short- to intermediate-duration evidence of multifunctionality, but remain preliminary for chronic polymicrobial and encrusting conditions.

A more sophisticated route uses the zwitterionic matrix as a responsive or self-defensive platform, such that bactericidal activity is activated only when the local environment signals infection. Representative examples include zwitterion-dominant coatings that become more bactericidal under acidic biofilm conditions^113^ (Figure 2C), and self-defensive PSBMA systems that couple antifouling to bacteria-triggered antibiotic release and localized surface shedding^114^ (Figure 2D). These designs are conceptually attractive because they recognize that long-term urinary catheter coatings may need to shift function over time rather than remain static. Their challenge, however, is that greater biological sophistication usually entails greater structural complexity and more demanding requirements for reliability under variable urinary conditions. Not all such systems have yet been validated on clinically realistic urinary catheter models, but together they define the main multifunctional directions now emerging in the field.

#### 2.4.4 Architecture, Thickness, and Durability

The hydration-stability trade-off becomes especially clear when zwitterionic coatings are compared as architectures rather than simply as chemistries. Recent work highlights a useful distinction between dense zwitterionic hydrogel reservoirs and thin surface-bound or brush-like coatings.^115,116^ This distinction is particularly important for urinary catheters because coating thickness affects not only lubricity and antimicrobial loading but also lumen preservation, surface conformity, and long-term mechanical tolerance. The central design question is therefore not whether hydrogels or brushes are intrinsically superior, but how thickness alters both the benefits and liabilities of a hydrated interface.

Dense zwitterionic hydrogels function as swollen three-dimensional carrier networks with high loading capacity and sustained or responsive release. Representative systems include sulfobetaine hydrogels incorporating silver-loaded metal–organic framework fillers for prolonged silver release^117^ (Figure 3A) and tannic-acid-containing PSBMA hydrogels that increase loading of iodine, copper, and nitrofurazone^118^ (Figure 3B). These systems illustrate the main strengths of hydrogel architectures: they are more tolerant of surface irregularity, better suited to high lubricity and interfacial compliance, and more capable of incorporating multifunctional payloads. Their weakness is equally consistent across the literature. Greater thickness increases swelling, softening, and interfacial mismatch, thereby increasing the durability burden under clinically relevant deformation and flow.

**Figure 3:**
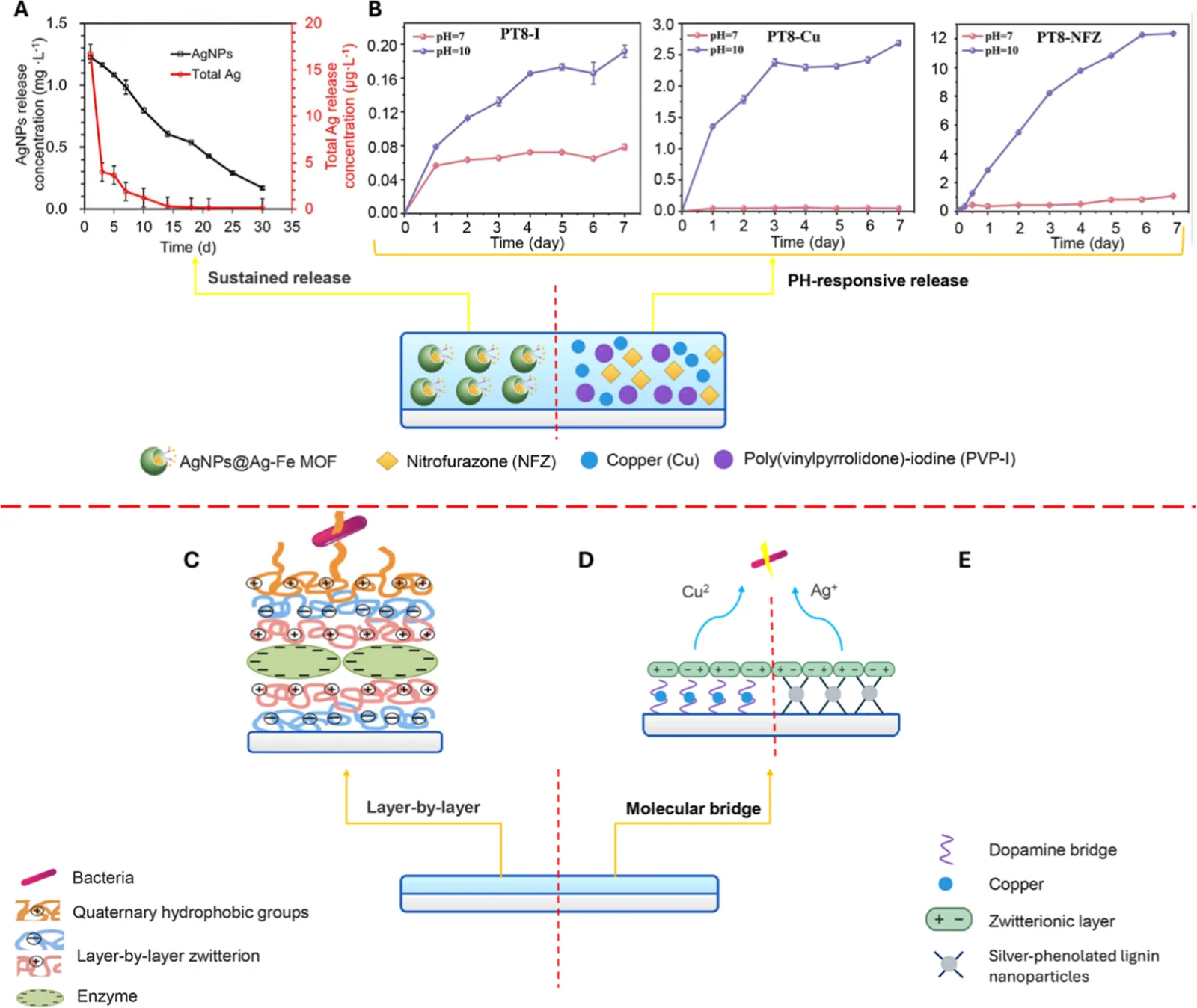
Architecture-dependent zwitterionic coating formats for urinary catheter applications. (A) AgNPs@Ag–Fe MOF-loaded sulfobetaine hydrogel coatings with high payload capacity and sustained silver release. Reproduced with permission from ref 117. Copyright 2025 Elsevier. (B) Tannic-acid-containing PSBMA hydrogel coatings with pH-responsive release of iodine, copper, and nitrofurazone. Reproduced with permission from ref 118. Copyright 2023 Royal Society of Chemistry. (C) Layer-by-layer polyzwitterion/enzyme coatings that confine antifouling and antibacterial functions within thin surface-bound interfacial layers. Reproduced from ref 68. Copyright 2016 American Chemical Society. (D) Polydopamine-assisted sulfobetaine/copper films formed through molecular-bridge-mediated surface anchoring. Schematic summary based on ref 119. (E) Laccase-assisted poly­(carboxybetaine) coatings incorporating silver-phenolated lignin nanoparticles for surface-confined antibacterial functionality. Schematic summary based on ref 70. The figure layout and annotations were assembled by the authors using Microsoft PowerPoint (Microsoft 365).

By contrast, thin interfacial coatings confine the zwitterionic component to a surface-bound film, multilayer, or brush-like architecture rather than a bulk hydrogel reservoir. Examples include layer-by-layer polyzwitterion/enzyme coatings, polydopamine-sulfobetaine/copper films, and laccase-assisted poly­(carboxybetaine) coatings incorporating silver-phenolated lignin nanoparticles^68,70,119^ (Figure 3C–E). These thin-layer systems are advantageous where preservation of catheter dimensions, flexibility, and lumen geometry is critical, making them especially relevant to the inner lumen.^120^ Their limitations arise from the same dimensional economy that makes them attractive, because reduced thickness usually means lower payload capacity and greater dependence on robust surface anchoring and coating uniformity. Moreover, many brush-like strategies have been developed on relatively smooth model substrates, whereas urinary catheter surfaces, particularly those formed from latex-based materials or with irregular inner luminal geometries, present much rougher and more heterogeneous interfaces.^63,121–123^

These studies suggest that coating thickness is not a secondary formulation variable, but a governing design parameter. Hydration-rich hydrogel coatings may be better suited to outer-surface lubrication, conformal coverage, and multifunctional loading, whereas thin brush-like or film-like coatings may be better matched to luminal applications where dimensional fidelity is critical.^13,61^ The most effective future urinary catheter designs will therefore likely arise not from selecting one architecture universally, but from matching coating format and thickness to the specific catheter interface being modified.

Table 2 places the available catheter-specific values within this architecture-dependent framework rather than using them as head-to-head rankings. In ref 65, an approximately 8 μm PSBMA coating reduced fibrinogen deposition by 72.3%, reduced *E. coli* and *Staphylococcus aureus* adhesion by >75%, and lowered the dynamic coefficient of friction from 0.63 to 0.35. Ref 66 illustrates the associated hydration–stability trade-off: PSBMA equilibrium swelling was approximately 510%, HPA incorporation reduced swelling by up to 91%, and static/dynamic coefficients of friction were 0.15/0.10 for PSBMA and 0.29/0.27 for PHPA. At a 4:1 SBMA/HPA ratio, the coating retained an approximately 40% reduction in fibrinogen deposition and an approximately 4-fold reduction in biofilm coverage relative to PDMS, with no visible delamination after 4 weeks in water.^65,66^ Because load, counterface, hydration state, conditioning medium, organism, and flow differ across the studies, these values should not be used to rank formulations directly.

These examples also expose important reporting gaps. Most catheter-specific studies do not report dry and hydrated thickness, swelling, quantitative coating–substrate adhesion, cyclic wear, and sterilization-aged friction in the same system. Although ref 67 demonstrated no delamination during repeated loading, stretching, torsion, and clamping, it did not report a standardized interfacial fracture energy.^67^ Thus, qualitative integrity should not be presented as quantitative adhesion strength. A more complete comparison would pair wet/dry thickness and swelling with peel, lap-shear, or fracture-energy measurements and cyclic friction under urine-conditioned, curved catheter geometry.

#### 2.4.5 Durability and Manufacturability

Once coating architecture is considered explicitly, the next question is how zwitterionic coatings retain antifouling, antibacterial, and lubricious function over clinically relevant time scales without mechanical failure. Most work on improving the robustness of zwitterionic materials has focused on bulk hydrogels rather than coatings,^124^ but that distinction is important because thin catheter coatings do not necessarily fail in the same way as thick, highly swollen layers. In thicker coatings, swelling, softening, and interfacial mismatch become dominant concerns, whereas in thinner systems the main vulnerabilities may instead be interfacial aging or insufficient anchoring density. In both cases, however, the central challenge is the same: to preserve a stable hydrated interface under deformation, shear, prolonged wet exposure, repeated insertion-withdrawal, and microbiological challenge.

One major response has been to strengthen interfacial adhesion.^125,126^ Studies on hydrogel-elastomer hybrids show that elastomeric substrates can inhibit radical polymerization, undergo hydrophobic recovery, and provide too few stable reactive sites for dense grafting.^127^ Recent zwitterionic work has therefore used strategies such as benzophenone-mediated grafting, polydopamine-assisted anchoring, and silane-assisted interfacial polymerization to improve bonding to silicone- or polyurethane-like substrates^67,125,127,128^ (Figure 4). Strong initial adhesion, however, does not guarantee long-term stability because siloxane-mediated bridges may hydrolyze in wet or alkaline environments,^129^ and dopamine-derived interlayers may age progressively.^130^ Interfacial adhesion should therefore be treated not simply as a deposition problem, but as a central durability challenge requiring both a sufficient density of stable anchoring sites and coupling bridges that can withstand hydration, salt exposure, swelling stress, and repeated deformation without progressive failure.

**Figure 4:**
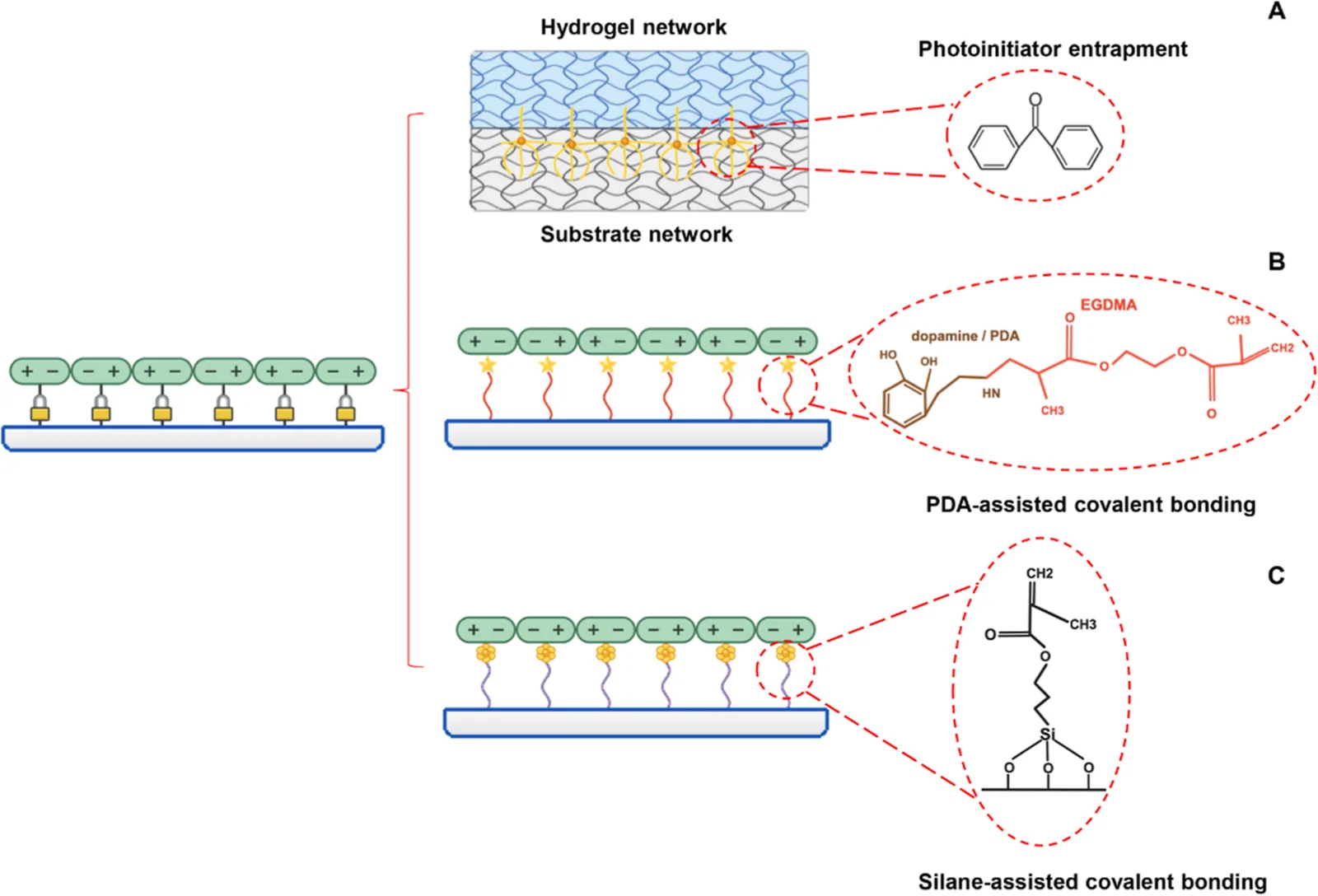
Interfacial anchoring strategies for enhanced delamination resistance of zwitterionic coatings on elastomeric catheter substrates. (A) Photoinitiator entrapment or photografting to generate interpenetrating or cross-linked hydrogel-substrate networks. (B) Polydopamine-assisted adhesive interlayers that introduce covalent bonding sites for zwitterionic coating formation. (C) Silane-assisted coupling that tethers zwitterionic layers to reactive substrate surfaces. Created by the authors using Microsoft PowerPoint (Microsoft 365).

A second response has focused on reinforcing the zwitterionic network itself through cross-link tuning and related structural strategies. Insufficient cross-linking compromises swelling control and mechanical integrity, whereas excessive cross-linking suppresses hydration and low-fouling performance.^131^ More elaborate approaches include micellar domains, hydrophobic comonomers, interpenetrating networks, and microgel reinforcements that restrain swelling while dissipating stress^66,132–135^ (Figure 5). Across these systems, the design objective is consistent: to retain hydration-mediated function while introducing sufficient structural support to prevent swelling-driven failure. Microgel reinforcement is especially attractive because the reinforcing microgels can be synthesized separately, tuned independently, and then blended into the base zwitterionic matrix. In the representative poly­(carboxybetaine) microgel-reinforced poly­(sulfobetaine) system reported by Yao and co-workers, PCB microgels served as reinforcing nodes, antiswelling components, and stress-dissipating domains within a continuous zwitterionic network, thereby enhancing coating toughness and durability without sacrificing hydration.^134^ Chai and colleagues extended this design logic further by developing a drug-loaded, temperature-responsive microgel-reinforced zwitterionic hydrogel catheter coating, in which the microgels reinforced the hydrogel, dissipated mechanical energy, strengthened interfacial bonding, and served as antibiotic carriers.^135^

**Figure 5:**
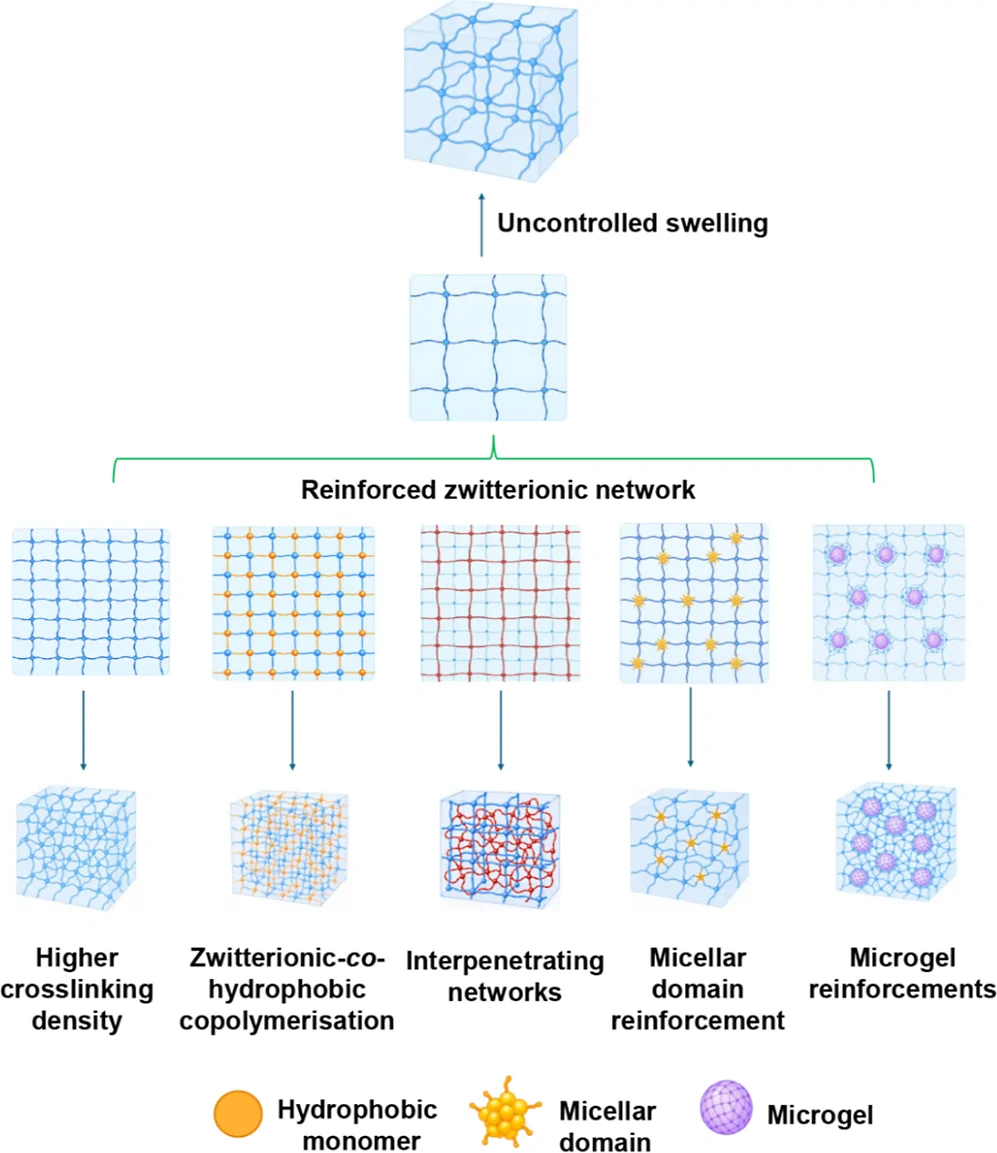
Structural reinforcement strategies for improving the network stability and antiswelling performance of zwitterionic hydrogels. Representative approaches include increasing cross-linking density, zwitterionic-hydrophobic copolymerization, construction of interpenetrating networks, and incorporation of micellar domains or microgels as independently tunable reinforcing nodes. Created by the authors using Microsoft PowerPoint (Microsoft 365).

A third response has been to move beyond static reinforcement toward adaptive durability. Self-healing and self-defensive zwitterionic systems are conceptually important because they shift the design objective from simple resistance to damage toward retention of function after damage.^114,136,137^ A representative example is the poly­(vinyl alcohol)/poly­(ethylene glycol)/quaternary ammonium compound hydrogel coating reported by Ran and co-workers, in which host–guest cross-linking between β-cyclodextrin and adamantane created a dynamic network capable of repeated dissociation and recombination.^137^ This design enabled rapid scratch healing, recovery of most mechanical properties, and tolerance of stretching, twisting, knotting, and compression while maintaining low friction and infection-responsive bactericidal behavior. Zhang and colleagues reported a conceptually related self-defensive PSBMA coating, in which interpenetration with the substrate improved coating integrity while bacteria-triggered degradation and renewal converted localized fouling into a regenerative process rather than irreversible surface failure.^114^ Although such systems remain early stage for urinary devices, they are important because they acknowledge that long-term catheterization is a dynamic environment involving repeated friction, deformation, hydration changes, and microbial challenge rather than a single fixed test condition.

#### 2.4.6 Sterilization, Storage, and Aging Stability

Durability during fresh immersion does not establish stability after sterilization or storage. Terminal sterilization should therefore be treated as a materials-processing step rather than a packaging detail. Heat can promote dehydration, chain rearrangement, or hydrolysis; ionizing radiation can generate chain scission or cross-linking; and oxidative plasma or ethylene oxide can alter surface chemistry, residuals, and interfacial bonding. Because direct comparative data are limited and architecture-specific, evidence from model systems must be interpreted cautiously. In sub-10 nm PEG, pSBMA, and pMPC coatings on silicon, Iqbal et al. found <5% thickness change for e-beam-treated pSBMA and EtO-treated pMPC, whereas H2O2 plasma decreased all coating thicknesses by at least 30%; protein adsorption increased for pMPC after every tested method. These model-film data show that preserved wettability or thickness alone does not guarantee preserved antifouling. However, they cannot be extrapolated directly to elastomeric catheters.^138^

A complementary bulk-material study provides a different perspective. In CB hydrogels, Han et al. reported retention of stress, modulus, and nonfouling performance after autoclave and ethylene oxide treatment, and improved gamma-irradiation tolerance when irradiation occurred in the dry rather than hydrated state.^139^ Although this demonstrates bulk-material feasibility, it does not establish coating–catheter adhesion. Accordingly, each candidate should be sterilized on the finished device using the intended dose, cycle, and package, then reassessed for chemistry, wet/dry thickness, swelling, coefficient of friction, adhesion/delamination during bending, torsion and cyclic insertion, particulates/extractables, and antimicrobial/antifouling function after urine conditioning.

Even when sterilization is tolerated, storage and aging can create a separate failure pathway through dehydration, hydrophobic recovery, catechol oxidation, siloxane hydrolysis, payload loss, or packaging interactions.^129,130^ Validation should therefore compare dry and prehydrated packaging where relevant and control temperature, humidity, light, and oxygen. It should combine accelerated and real-time aging, quantify extractables and hydration recovery, and repeat the full simulated-use dwell test at the end of shelf life. On this basis, a coating should be described as durable only when it retains function across sterilization, shelf life, activation, and intended use, rather than only during fresh immersion.

#### 2.4.7 Translational Roadmap

From a translational perspective, these advances now need to be organized more explicitly around device-development criteria rather than chemistry alone. A useful translational roadmap is to evaluate each coating across six domains: interfacial performance, biocompatibility, sterilization compatibility, storage and packaging stability, durability after deformation and aging, and labeled performance during indwelling use under clinically realistic conditions. Framing the field in this way shifts the key question from “Does the coating work *in vitro*?” to “Can the coating remain functional across the product lifecycle?” ^72–74^ Future progress will therefore depend not only on more effective materials but also on more predictive validation models that integrate host-protein conditioning, dynamic urine flow, urethral friction, polymicrobial challenge, and mineral deposition within the same framework.^13,16,46–49^ It will also depend on coating chemistries that can survive sterilization, remain stable through storage and transport, and retain functionality across the full labeled dwell period rather than only in short laboratory assays.^71–74^ In this respect, the key challenge is no longer simply to make zwitterionic coatings more antifouling, but to translate hydration-mediated interfacial design into manufacturable, durable, and clinically translatable urinary catheter technologies.

### 2.5 Evidence-Based Design Rules and Remaining Uncertainty

Viewed across these studies, the current evidence is best interpreted in tiers. The most consistent findings are hydration-mediated reductions in protein adsorption and initial bacterial adhesion and lower friction in controlled catheter-relevant models. By comparison, evidence for antiswarming copolymers, contact-killing or responsive composites, week-scale suppression of mineralization, and mechanically reinforced or self-healing coatings remains promising but preliminary because these strategies rarely combine polymicrobial urine flow, deformation, sterilization, aging, and clinically matched dwell in one study.^64–66,69,70,114^ At the clinical level, the reported human outcome data are encouraging, but they are available only as interim conference material for one proprietary platform and do not identify a superior zwitterionic chemistry.^71–75^

These evidence tiers lead to five design rules: (1) define the target interface and intended dwell period before selecting chemistry; (2) treat early antiadhesion, prolonged preclinical protection, and clinical benefit as separate claims; (3) select PC, SB, or CB together with architecture and anchoring because chemistry alone does not determine stability; (4) optimize multifunctional cationic content against conditioning-dependent antifouling and biocompatibility rather than maximizing bactericidal charge; and (5) define durability across sterilization, storage, mechanical cycling, urine flow, polymicrobial challenge, and mineralization. Together, these rules shift assessment from isolated fresh-coating performance to retention of function across the catheter product lifecycle.

## 3 Conclusion

CAUTI remains a major and unresolved complication of urinary catheterization, reflecting the persistent limitations of current catheter materials and surface technologies. As this review has shown, catheter failure is not driven by infection alone, but by a coupled interfacial process involving tissue trauma, host-protein conditioning, biofilm formation, bacterial migration, and, in long-term use, encrustation and blockage. In this setting, zwitterionic coatings offer a compelling biomaterials strategy because their strongly hydrated surfaces can reduce nonspecific protein adsorption while also supporting lubrication, biocompatibility, and multifunctional surface engineering.

Recent studies suggest that these coatings can be tailored to address urinary catheter-specific challenges, including fibrinogen deposition, bacterial swarming, persistent biofilm formation, and encrustation, while advances in coating architecture and interfacial anchoring are beginning to improve mechanical durability. However, the evidence remains largely preclinical. The available human outcome data are interim, conference-reported, and product-specific, so no zwitterionic coating can yet be regarded as an established solution for long-term catheterization. Future progress should therefore focus on scalable manufacturing of durable coatings, standardized testing under clinically realistic urinary conditions, and rigorous clinical studies that evaluate long-term efficacy, safety, and device performance. Ultimately, successful translation will depend not on identifying a single ideal zwitterionic chemistry, but on engineering catheter-specific interfaces that balance antifouling activity, lubrication, durability, manufacturability, and clinical relevance. To support that translation, future studies should prespecify the intended dwell time and distinguish initial-interface, prolonged preclinical, and clinical end points.
